# Analysis of Chemokines and Receptors Expression Profile in the Myelin Mutant *Taiep* Rat

**DOI:** 10.1155/2015/397310

**Published:** 2015-03-25

**Authors:** Guadalupe Soto-Rodriguez, Juan-Antonio Gonzalez-Barrios, Daniel Martinez-Fong, Victor-Manuel Blanco-Alvarez, Jose R. Eguibar, Araceli Ugarte, Francisco Martinez-Perez, Eduardo Brambila, Lourdes Millán-Perez Peña, Nidia-Gary Pazos-Salazar, Maricela Torres-Soto, Guadalupe Garcia-Robles, Constantino Tomas-Sanchez, Bertha Alicia Leon-Chavez

**Affiliations:** ^1^Facultad de Ciencias Químicas, Benemérita Universidad Autónoma de Puebla, 14 Sur y Avenida San Claudio, 72570 Puebla, PUE, Mexico; ^2^Laboratorio de Medicina Genómica, Hospital Regional 1° de Octubre, ISSSTE, Avenida Instituto Politécnico Nacional No. 1669, 07760 México, DF, Mexico; ^3^Departamento de Fisiología, Biofísica y Neurociencias, Centro de Investigación y de Estudios Avanzados del Instituto Politécnico Nacional, Apartado Postal 14-740, 07000 México, DF, Mexico; ^4^Instituto de Fisiología, Benemérita Universidad Autónoma de Puebla, 14 Sur 6301, 72560 Puebla, PUE, Mexico; ^5^Laboratorio de Genómica de Celomados, Grupo de Microbiología y Genética de la Escuela de Biología, Universidad Industrial de Santander, Apartado Postal 680002, Bucaramanga, Colombia

## Abstract

*Taiep* rat has a failure in myelination and remyelination processes leading to a state of hypomyelination throughout its life. Chemokines, which are known to play a role in inflammation, are also involved in the remyelination process. We aimed to demonstrate that remyelination-stimulating factors are altered in the brainstem of 1- and 6-month-old *taiep* rats. We used a Rat RT^2^ Profiler PCR Array to assess mRNA expression of 84 genes coding for cytokines, chemokines, and their receptors. We also evaluated protein levels of CCL2, CCR1, CCR2, CCL5, CCR5, CCR8, CXCL1, CXCR2, CXCR4, FGF2, and VEGFA by ELISA. Sprague-Dawley rats were used as a control. PCR Array procedure showed that proinflammatory cytokines were not upregulated in the *taiep* rat. In contrast, some mRNA levels of beta and alpha chemokines were upregulated in 1-month-old rats, but CXCR4 was downregulated at their 6 months of age. ELISA results showed that CXCL1, CCL2, CCR2, CCR5, CCR8, and CXCR4 protein levels were decreased in brainstem at the age of 6 months. These results suggest the presence of a chronic neuroinflammation process with deficiency of remyelination-stimulating factors (CXCL1, CXCR2, and CXCR4), which might account for the demyelination in the *taiep* rat.

## 1. Introduction

The* taiep* rats exhibit hypomyelination and suffer progressive demyelination resulting in a highly hypomyelinated central nervous system (CNS) as they reach adulthood [1, 2]. This demyelinating process has been associated with nitrosative stress* in vivo* and* in vitro* [3, 4], glial-microglial activation and lymphocyte migration [4], and an increase in lipoperoxidation, caspase-3 activation, and cell death* in vivo* [5]. The* taiep* rat has been considered as a chronic animal model of multiple sclerosis (MS) [6], which is characterized by a remyelination failure in areas of chronic demyelination with absence of oligodendrocyte progenitor cells (OPCs) and acute inflammation [7]. It has been suggested that astrogliosis in a microenvironment of chronic inflammation forms a barrier that impedes the OPC migration [7]. However, induction of an acute inflammation in areas of chronic demyelination can activate remyelination as proven in the* taiep* rat [8]. These results suggest that OPCs in the* taiep* rat are unable to generate myelinating oligodendrocytes due to the lack of the stimulatory factors and/or the presence of inhibitory factors [8].

Previous studies in the* taiep* rat have shown glial cell priming [3], reactive astrogliosis since 1 month of age [9], and lymphocyte infiltration at 6 months of age [4], suggesting the participation of glial cells in the early age and leukocytes in the adulthood. To date, the immunological mediators involved in those differential cell processes in the* taiep* rat remain unknown. Several reports in different animal models [10–15] and in human patients with MS [16] sustain the involvement of chemokines and their receptors in the CNS inflammation. Alternatively, chemokines also participate in myelin development. CXCL1 has been shown to play an important role in proliferation, differentiation, migration, and maturation of oligodendroglial cells and myelin synthesis [17–19]. Moreover, the deficit of CXCL1 and CXCR2 causes failure in the myelination process due to an aberrant migration of OPCs in the spinal cord white matter [20]. CXCL12 activating CXCR4 receptor has been involved in the OPCs maturation and remyelination failure [21]. CXCL12 also acts as a growth factor for stimulating the astrocyte proliferation and neuronal cells [22, 23], via extracellular signal-regulated kinases (ERK 1/2) [24].

Based on the finding that the remyelination process is absent in the* taiep* rat [7, 8], it is important to determine chemokines and their receptors especially those that act as remyelination-stimulating factors. The present work aimed to evaluate whether a deregulation of chemokine expression profile occurs in the* taiep* rat. We used RT^2^ Gene Profiler PCR Array to assess cytokines, chemokines, and their receptors and ELISA to measure CCL2, CCR2, CCL5, CCR5, CXCL1, CXCR2, and CXCR4. The measurements were made in the brainstem from 1- to 6-month-old* taiep* rats because this is the second region most affected and presents atypical cells at those ages [4]. The results were compared with those in Sprague-Dawley (SD) rats. Our results showed the deficiency of CXCL1 and CXCR4 levels in the* taiep* rats, suggesting that these chemokines might be involved in the remyelination failure in those animals.

## 2. Materials and Methods

### 2.1. Experimental Animals

*Taiep* rats of different ages (one and six months old) were obtained from the vivarium of the Institute of Physiology, BUAP. Sprague-Dawley (SD) rats (negative controls) were supplied by CINVESTAV vivarium. Institutional Animal Care and Use Committee (IACUC) approved our animal use procedures with the protocol number 410-08. Animals were maintained in rooms with controlled conditions of temperature (22 ± 1°C) and light-dark cycle (12 : 12 h light : dark; light onset at 07:00). Food and water were provided* ad libitum*. All procedures were in accordance with the Mexican current legislation, the NOM-062-ZOO-1999 (SAGARPA), based on the Guide for the Care and Use of Laboratory Animals, NRC. All efforts were made to minimize animal suffering.

### 2.2. PCR Array

The total RNA (1.0 *μ*g) extracted from 1- to 6-month-old brainstem of SD and* taiep* rats was quantified with a NanoDrop Spectrophotometer (Thermo Scientific NanoDrop Technologies, Wilmington, DE, USA). Reverse transcription reaction was made with the RT^2^ PCR Array First Strand Kit from SABiosciences (Qiagen Company). Real-time PCR was conducted on a 384-well plate for Chemokines and Receptors RT^2^ Profiler PCR Array of Rat (PARN-022Z, Qiagen), which contains a profile for the expression of 84 genes that encode chemokines and their receptors and cytokines. The amplification assays were made using a 7900HT Fast Real-Time PCR System (Applied Biosystems, Foster City, CA, USA) (http://www.sabiosciences.com/PCRArrayPlate.php).

### 2.3. Enzyme-Linked Immunosorbent Assay (ELISA)

ELISA was used to determine the protein levels of CCL2, CCR1, CCR2, CCL5, CCR5, CCR8, CXCL1, CXCR2, CXCR4, FGF2, and VEGFA in homogenates obtained from the brainstem of SD or* taiep* rats at 1 and 6 months of age (*n* = 5 per age in each group), as described previously [4]. The protein content was determined using the method by Sedmak and Grossberg [25]. Aliquots containing 5 *μ*g of total protein were placed into wells of ELISA plates to determine chemokines and receptors in separate assay. Then, proteins were denatured by the addition of 100 *μ*L of 0.1 M carbonate buffer (pH 8.0) added into each well and the plate was incubated for 18 h at 4°C. To block nonspecific binding sites, 200 *μ*L of 0.5% bovine serum albumin, IgG-free, was added to each well at room temperature (RT). After 30 min of incubation, the wells were washed thrice with PBS-Tween 20 (0.1%). Rabbit monoclonal antibodies to CCL2, CCR1, CCR2, CCL5, CCR5, CCR8, CXCL1, CXCR2, CXCR4, FGF2, and VEGFA (1 : 500 dilution, Abcam Inc., Cambridge, MA, USA) detect both native and denatured proteins. They were added into each well and incubated for 2 h at RT. After three washings with PBS, a horseradish peroxidase-conjugated goat anti-rabbit IgG (1 : 1000 dilution, Dako North America Inc., Carpinteria, CA, USA) was added and incubated for 2 h at RT. The antigen-antibody complex was revealed by adding 100 *μ*L of 2,2′-azino-bis(3-ethylbenzthiazoline-6-sulfonic acid) (ABTS) containing 0.3% H_2_O_2_ into each well. After 15 min, the optical density (OD) was determined at 415 nm using a Benchmark multiplate reader (Bio-Rad, Hercules, CA, USA) as was described elsewhere [4].

### 2.4. Indirect Immunofluorescence

CCR2, CCR5, CXCR2, and CXCR4 were detected in brain sagittal slices of 6-month-old SD and* taiep* rats (*n* = 3 per each group). Rats were deeply anesthetized with chloral hydrate and perfused through the ascending aorta with 100 mL of PBS 1x and then by 150 mL of 4% paraformaldehyde in PBS. Their brains were removed and maintained in the fixative for 48 h at 4°C. Each brain was included in paraffin and was cut into 3 *μ*m slices on the sagittal plane using a Leica RM 2135 microtome (Leica Microsystems, Nussloch, Germany). Slices were individually collected on a glass slide. Tissue slices (previously deparaffinized) were rehydrated and incubated with 0.5% IgG-free bovine serum albumin in PBS-Tween 20 (0.1%) for 20 min at RT. Slices were incubated with rabbit monoclonal antibodies to CCR2, CCR5, CXCR2, or CXCR4 (1 : 500 dilution, Abcam Inc., Cambridge, MA, USA) at 4°C overnight. The secondary antibody was a goat anti-rabbit IgG conjugated with fluorescein isothiocyanate (FITC). The counterstaining was made using DAPI or propidium iodide (2 *μ*g/mL). Tissue slices were mounted on glass slides using VECTASHIELD (Vector Laboratories, Burlington, Ontario, Canada). The fluorescence within the cells was analyzed with 5x and 40x objectives of a Leica DMIRE2 microscope using the filters A for DAPI, K3 for FITC, and TX2 for propidium iodide (Leica Microsystems, Wetzlar, Germany). The images were digitalized with a Leica DC300F camera (Leica Microsystems, Nußloch, Germany) and processed with a workstation Leica FW4000, version V1.2.1 (Leica Microsystems Vertrieb GmbH, Bensheim, Germany). Samples incubated with only the secondary antibodies were used as negative controls.

### 2.5. Statistical Analysis

All values are provided as the mean ± standard error of the mean (SEM). The difference between the groups with respect to age in all the assays was analyzed using a one-way ANOVA test. The differences between SD and* taiep* rats were determined with unpaired Student's *t*-test. All statistical analyses were performed using the GraphPad Prism software (GraphPad Software Inc., San Diego, CA, USA). *P* values < 0.05 were considered significant. Analysis of qPCR data was performed based on a web-based PCR Array Data Analysis protocol (http://pcrdataanalysis.sabiosciences.com/pcr/arrayanalysis.php) provided by SABiosciences (Qiagen) and the results are expressed as a fold change.

## 3. Results

**Figure 1:**
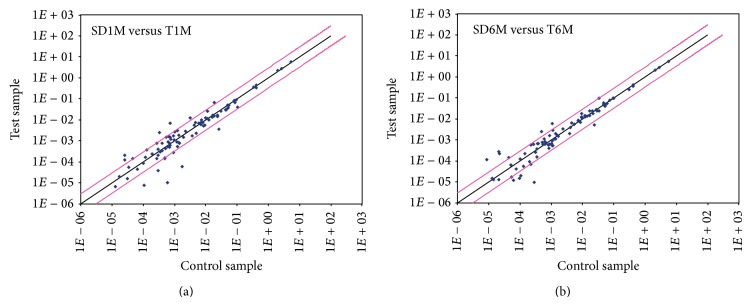
Overview of scatter plot of expression of 84 genes. Analysis of expression of genes from brainstem of* taiep* rats compared with Sprague-Dawley rats by RT^2^ Profiler PCR Array. The black line indicates fold changes [2^(−Δ*C*_*t*_)^] of 1. The pink lines indicate the desired fold change in gene expression threshold, defined by the user with the entry in cell A1. SD1M = 1-month-old Sprague-Dawley (SD) rats; T1M = 1-month-old* taiep* rats; SD6M = 6-month-old SD rats; T6M = 6-month-old* taiep* rats.

**Figure 2:**
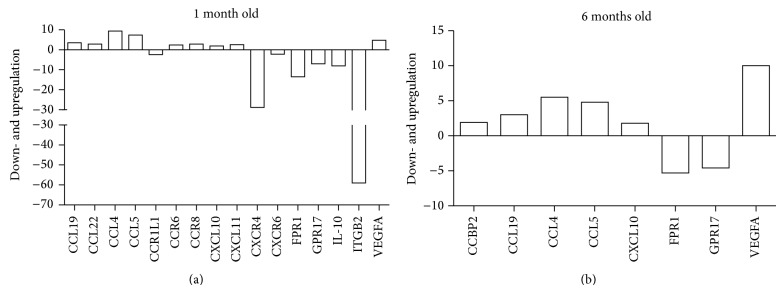
Chemokine expression profile in the brainstem of* taiep* rats 1 and 6 months old. Deregulation in chemokines and chemokine receptors was determined by RT^2^ Profiler PCR Array in the brainstem of* taiep* rats one and six months old. Values show fold up- or downregulation as compared to control group, Sprague-Dawley rats at the same age.

The scatter plots show the expression profile of 84 genes of* taiep* rat in comparison with the control profile of SD rats (Figure 1). No upregulation of proinflammatory cytokines was determined at any age and group studied. In contrast, downregulation was established for IL-1*β* (−3.3, *P* < 0.05) and IL-10 (−8.0, *P* < 0.05) at 1-month-old, ITG-*β*2 at both 1- (−59.3, *P* = 0.01) and 6-month-old (−5.4, *P* = 0.01), and MAPK1 at 6-month-old (−29.9, *P* = 0.01)* taiep* rats (Figure 2). Downregulation was also established for inflammation mediators such as formyl peptide receptor 1 (FPR1) at 1- (−13.5, *P* < 0.05) and at 6-month-old (−5.31, *P* < 0.05) and GPR17 at 1- (−7.03, *P* = 0.05) and at 6-month-old (−4.6, *P* < 0.05)* taiep* rats. These results show that* taiep* rat brainstem exhibits a decrease in inflammatory and anti-inflammatory cytokine.

Expression of some chemokines and their receptors was modified in the brainstem of* taiep* rat when compared with those in SD rat. Gene upregulation of CCL19 at 1- (3.5, *P* = 0.01) and at 6-month-old (3.0, *P* = 0.05), CCL22 at 1-month-old (2.8, *P* = 0.005), CCL4 at 1-month-old (9.3, *P* = 0.003), CCL5 at 1- (7.4, *P* = 0.02) and at 6-month-old (4.8, *P* = 0.05), and CCR6 at 1-month-old (2.4, *P* = 0.05)* taiep* rats was found. Alpha chemokines were also upregulated; CXCL10 at 1- (1.9, *P* = 0.02) and at 6-month-old (1.8, *P* = 0.004), CXCL11 at 1-month-old (2.6, *P* = 0.003), and VEGFA at 1- (4.8, *P* < 0.05) and at 6-month-old (10, *P* = 0.03) (Figure 2)* taiep* rats were found.

CCR1L1 was downregulated at 1-month-old (−2.36, *P* = 0.02), CCR8 at 1-month-old (−6.6, *P* = 0.03), and CXCR4 at 1-month-old (−28.8, *P* < 0.05), at 6-month-old (−5.3, *P* < 0.05), and at 6-month-old (−2.2, *P* = 0.01)* taiep* rats.

**Figure 3:**
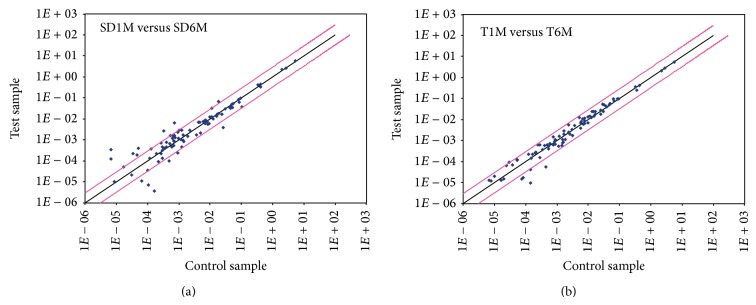
Overview of scatter plot on expression of 84 genes. Analysis of gene expression in the brainstem at different ages of Sprague-Dawley and* taiep* rats by RT^2^ Profiler PCR Array. The black line indicates fold changes [2^(−Δ*C*_*t*_)^] of 1. The pink lines indicate the desired fold change in gene expression threshold, defined by the user with the entry in cell A1. SD1M = 1-month-old Sprague-Dawley (SD) rats; T1M = 1-month-old* taiep* rats; SD6M = 6-month-old SD rats; T6M = 6-month-old* taiep* rats.

Comparisons between results from 6-month-old versus 1-month-old rats in the same group show no significant changes in gene expression in SD rats, except for two downregulated chemokines, CXCL9 (−3.9, *P* < 0.05) and TGFB1 (−3.6, *P* < 0.05), while, in* taiep* rats, gene expression was downregulated for 5 chemokines: CCR8 (−5.2, *P* = 0.05), CCR9 (−7.7, *P* < 0.05), CXCL5 (−2.2, *P* < 0.05), MAPK1 (−15.3, *P* < 0.05), and TGFB1 (−3.5, *P* < 0.05) (Figure 3).

**Figure 4:**
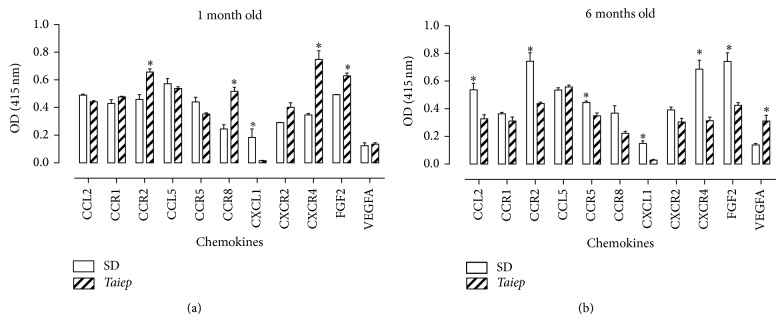
Protein levels of chemokines and their receptors in the brainstem of Sprague-Dawley (SD) and* taiep* rats. Chemokines, receptors, and growth factors were assayed using indirect ELISA. Each value represents the mean ± SEM of 5 independent experiments made in triplicate. ^∗^Significantly different from SD rats (Student's* t*-test). The significance was established at *P* < 0.05.

Comparison of protein levels between* taiep* rats and SD rats showed changes in chemokine receptors and growth factors at both ages. At 1 month of age, receptors that increased were CCR2 (43.5 ± 5.1%), CCR8 (111.3 ± 12.4%), CXCR4 (115.8 ± 18.1%), and FGF2 (27.6% ± 4.1). In contrast, CCR5 (−19.8 ± 5.1) and CXCL1 (−82.2 ± 1.6%) were decreased (Figure 4). In the brainstem of 6-month-old rats, protein levels were decreased for CCL2 (−39.0% ± 5.5%), CXCL1 (−80.9% ± 3.6%), CCR1 (−14.4% ± 7.9%), CCR2 (−41.2% ± 1.4%), CCR5 (−21.8% ± 5.8%), CCR8 (−34.6% ± 5.8%), CXCR4 (−54.4% ± 3.7%), and, the growth factor, FGF2 (−42.7% ± 2.7%). In contrast, VEGFA (125.5% ± 29.7%) levels increased (Figure 4).

**Figure 5:**
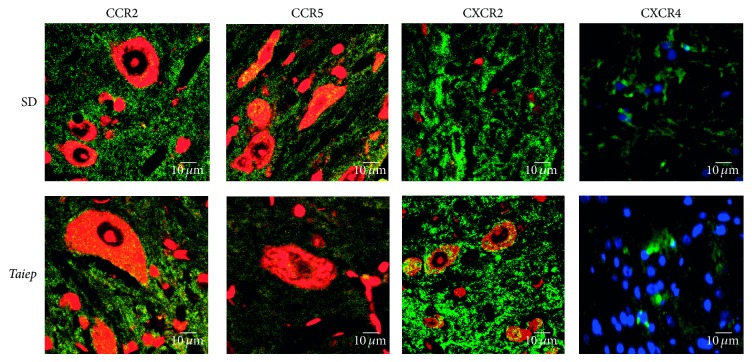
Immunoreactivity against receptors in the brainstem of* taiep* and Sprague-Dawley rats 6 months old. Paraffin-included slices of 3 *μ*m were immunostained with rabbit monoclonal anti-CCR2, anti-CCR5, anti-CXCR2, and anti-CXCR4 (fluorescein, green). Propidium iodide (red) or DAPI (blue) was used as nuclear counterstaining. SD; Sprague-Dawley rats.

Immunofluorescence studies showed decreased intensity against CCR5 and CXCR4 in the brainstem of 6-month-old* taiep* rats in comparison with SD rats. CCR5 immunostaining was evident in glial cells located in the white matter of both SD and* taiep* rats. CCR2 and CXCR2 were found in glial cells and neurons, but CXCR4 was observed in glial cells (Figure 5). CXCR2 immunofluorescence intensity in* taiep* rats was not different from that in SD rats (Figure 5).

## 4. Discussion

Results obtained in this work support the idea that the* taiep* rat exhibits a chronic neuroinflammatory profile that is different from other models of demyelination such as EAE. The major differences of* taiep* rats in comparison with EAE are that cytokines such as IFN-*γ*, TNF-*α*, and IL-6 were unaltered but IL-1*β* and IL-10 were downregulated in 1-month-old* taiep* rats. In normal pups, the exposure to stress stimuli leads to decreased levels of IL-10 and IL-1*β* in serum [26]. Downregulation of IL-1*β* and IL-10 in the brainstem of* taiep* rats might be explained in part by the motor alterations of* taiep* mother and pups, acting as a stress stimulus. In addition, downregulation of TGF-*β*1 was found in* taiep* rats. As TGF-*β*1 suppresses inflammation and promotes neuronal survival in adult CNS, the lack of these effects could be associated with the increased neuronal apoptosis and necrosis in the brain of adult* taiep* rats [5]. The decrease in TGF-*β*1 expression at 6-month-old* taiep* and SD rats might also be a sign of CNS aging.

We found that GPR17 was downregulated in the* taiep* rats. This G coupled protein receptor is an important mediator of OPC differentiation and white matter repair that is expressed by a subset of OPCs to operate as an early sensor of brain damage [27]. Accordingly, inhibition of GPR17 expression causes impairment in oligodendrocytes differentiation and myelination in* in vivo* and* in vitro* systems [28]. Supporting this, GPR17 downregulation could contribute to alterations in remyelination in the* taiep* rats. Another coupled protein receptor that was downregulated in the brainstem of 1-month-old* taiep* rats was FPR1 (formyl peptide receptor 1), which mediates the chemotaxis, activation, and cytokine gene transcription in phagocytic leucocytes in response to bacterial formylated chemotactic peptides [29]. These findings suggest that the immune response is decreased in the* taiep* rat, producing a predominant chronic neuroinflammatory process or astrocyte priming as was reported previously in cell culture [3].

CCL19 is a chemokine involved in the maintenance of chronic neuroinflammation [30, 31]. Accordingly, this chemokine was upregulated in the brainstem of* taiep* rats. The CCR8 mRNA and protein were also upregulated at the age of 1 month in the* taiep* rats. This finding can explain the accumulation of microglial cells in the CNS of* taiep* rats reported previously [4] due to the action of promoting chemotaxis of mononuclear cells and phagocytosis [32]. However, CCR8 protein levels were decreased in 6-month-old* taiep* rats, suggesting decrease in the infiltration of macrophages.

Although CCL5 was upregulated in the* taiep* rats at the age groups studied, protein levels were decreased in the brainstem, as well as its receptor CCR5 in 6-month-old rats. The reduction of CCR5 in mice prevents macrophage infiltration and demyelination [33, 34] and lack of CCL5 decreases neuronal survival [33, 35]. The decrease in CCL5 and CCR5 supports the cell death that occurs in* taiep* rats [5].

CXCL10 is involved in the T cell trafficking in the MS [36, 37]. Accordingly, upregulation of CXCL10 might explain the presence of CD 4(+) cells in the CNS of* taiep* rats reported previously [4].

The deficiency of the CXCL1 and CXCR4 proteins in the* taiep* rats might affect the proliferative response and recruitment of OPC to the injured areas [21, 38]. Lack of CXCL1 may also contribute to differentiation failure of OPCs [39, 40]. Altogether, these alterations of OPCs limit the remyelination in the* taiep* rats. On the contrary, the increased CXCL1 mRNA and protein levels in other myelin mutants such as jimpy mice were associated with a remyelination process in the spinal cord [41]. However, CXCL1 mRNA was not affected in the* taiep* rats; the decrease of CXCL1 protein levels could be explained by instability of mRNA or a failure in its regulation. Related to the former suggestion, it has been reported that CXCL1 mRNAs contain adenine-uridine-rich sequence elements (AREs) in their 3′-untranslated regions (3′-UTRs) that exhibit constitutive instability. These regions are stabilized by acute proinflammatory stimulus, that is, IL-17 [42–44], that is not present in the* taiep* rats.

The 1-month-old* taiep* rat showed difference between mRNA and protein CXCR4 that might be due to two causes: (1) a decrease in stabilization of CXCR4 mRNA or an increase in CXCR4 protein translation that would rapidly consume CXCR4 mRNA [45] and (2) the increase of CXCR4 protein in the brainstem that could be due to the presence of other CXCR4 positive cells such as OPCs that are known to migrate from ventral ventricular zone during myelination, which is still active at one month of age [46]. Supporting the latter suggestion is the finding that mRNA and protein CXCR4 are decreased in the brainstem of 6-month-old* taiep* rats, when the migration of OPCs was completed [47].

We found that VEGF mRNA and protein levels are increased in 6-month-old* taiep* rats. This factor released by astrocytes is known to disrupt the blood-brain barrier in CNS inflammatory disease [48]. In addition, VEGF promotes OPC migration to the myelination areas [49]. However, OPC migration is altered in* taiep* rats [7, 8] despite the increased VEGF levels. The alteration of OPC migration might be explained by the deficiency of CXL1 and CXCR4 found here. The role of VEGF in OPCs migration was proven by blocking VEGF receptor with anti-Flk-1 antibody [49]. Finally, the increased levels of VEGF, CXCL10, and CCL19 might account for the infiltration of lymphocytes CD4+ and CD8+ previously found in the* taiep* rats at the age of 6 months.

## 5. Conclusion

The mutation in the* taiep* rats that causes the microtubule accumulation in the oligodendrocyte and the consequent hypomyelination is still unknown [1, 2, 50]. Our results strongly suggest that the deficiency of the remyelination-stimulating factors such as CXCL1 and CXCR4 contributes to the failure in the process of remyelination in a microenvironment of chronic inflammation in the* taiep* rats. Further studies are needed to identify signal pathways and other mediators involved in the inflammatory response of the* taiep* rat.
